# Post-operative gastric outlet obstruction of giant hiatal hernia repair: a case report

**DOI:** 10.1186/s12876-022-02117-z

**Published:** 2022-02-05

**Authors:** ZhaoPeng Li, FuJia Xie, Lin Zhu, Liang Sun

**Affiliations:** Department of Gastrointestinal and Hernia Surgery, First Affiliated Hospital of Kunming Medical University, 295 Xichang Road, Wuhua, Kunming, 650032 Yunnan Province China

**Keywords:** Giant hiatal hernia, Complications, Laparoscopy, Surgery, Case report

## Abstract

**Background:**

Giant hiatal hernia is defined as those with more than 30% of the stomach herniating into the chest cavity. The transabdominal laparoscopic approach is the well-established repair form for giant hiatal hernia. To our best knowledge, reports on post-operative gastric outlet obstruction of giant hiatal hernia repair have been scanty up till now.

**Case presentation:**

A 45-year-old female patient was referred to the Emergency Department of our hospital with a chief complaint of acute right epigastric pain for 2 days. Physical examination revealed mild tenderness in the right epigastrium, without rebound tenderness or guarding. The abdominal computed tomography scan revealed a large low-density gastric artifact in the lower mediastinum—giant hiatal hernia. The barium swallow esophagogram and gastroscopy also confirmed the presence of a giant hiatal hernia. A transabdominal laparoscopic operation for reduction of the hernia contents and repair of the hiatal defect was performed. Her right epigastric pain alleviated obviously on the first postoperative day. On post-operative day five, however, she was presented with nausea and vomiting independent of meals. The nasogastric tube was inserted and kept in the stomach for 7 days. After removing the nasogastric tube, severe nausea and vomiting of the patient occurred again. Barium swallow revealed gastroptosis and enfoldment in the duodenal bulb, which indicated the presence of gastric outlet obstruction. Gastrojejunostomy was performed for her to relieve the gastric outlet obstruction. The patient was discharged on the tenth day after the second operation without any discomfort. During the regular follow-up period, she felt well and was satisfied with her status.

**Conclusions:**

Facing the giant hiatal hernia repair, the reduction of the hernia contents and repair of the hiatal defect being well operated on are insufficient, and we must watch out the anatomical variation, like the deviation of partial intra-abdominal organs from their normal positions, as well as paying attention to the protection of abdominal vagal nerve during the operation. Post-operative gastric outlet obstruction of giant hiatal hernia repair is rare, while gastrojejunostomy can successfully relieve the gastric outlet obstruction.

## Background

The definition of hiatal hernia is the prolapse of the stomach or other intra-abdominal organs through the hiatus of the diaphragm into the thoracic cavity. Giant hiatal hernia is defined as those with more than 30% of the stomach herniating protruding into the thoracic cavity [1, 2]. Giant hiatal hernia accounts for approximately 5–10% of all hiatal hernias [3]. The giant hiatal hernias with symptoms usually require surgical intervention. Laparoscopic hiatal hernia repair is currently preferred by surgeons.

Post-operative gastric outlet obstruction of giant hiatal hernia with the transabdominal laparoscopic repair is extremely rare. To our best knowledge, reports on post-operative gastric outlet obstruction of giant hiatal hernia repair have been scanty up till now, let alone the measures to solve it. Herein, we presented a female patient with a giant hiatal hernia who underwent transabdominal laparoscopic hernia repair and then was found to have post-operative gastric outlet obstruction. However, the obstructive symptoms disappeared after the operation of gastrojejunostomy.

## Case presentation

A 45-year-old female patient was referred to the Emergency Department of our hospital complaining of acute right epigastric pain, which lasted for 2 days. Her right epigastric pain was not alleviated obviously with the conservative treatments at the local hospital. She denied episodes of acid reflux, nausea, vomiting, dysphagia, or weight loss. She had a body-mass-index (BMI) of 20 kg/m^2^.

**Fig. 1:**
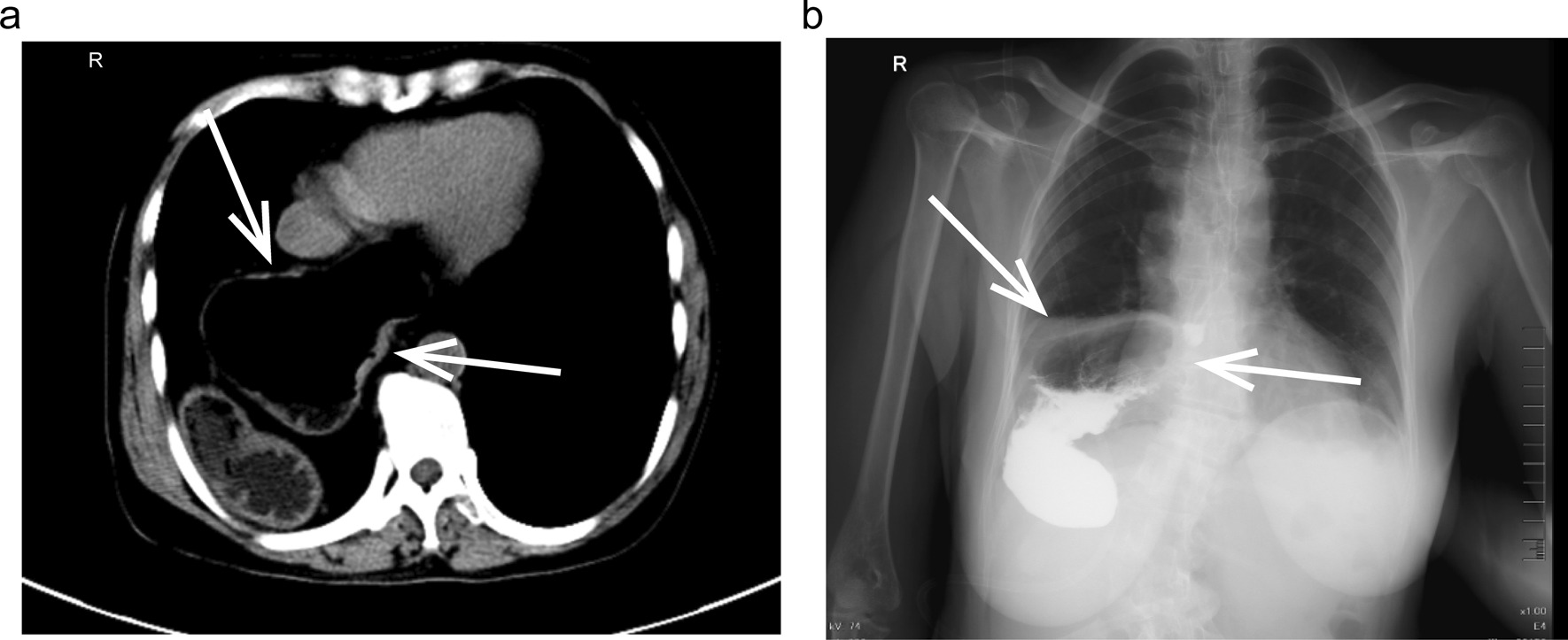
Pre-operative imaging evaluations of the patient. **a** CT-scan of the abdomen exhibiting the giant hiatal hernia (white arrows). **b** Barium swallow showing the giant hiatal hernia (black arrows)

Physical examination revealed mild tenderness in the right epigastrium, without rebound tenderness or guarding, and the rest of the abdomen was normal. No abnormal alterations in vital signs or electrocardiograms were detected. Routine blood investigations revealed white blood cells 7.9 × 10^9^/L, neutrophils (75.5%), lymphocytes (18.5%), and the red blood cell and platelet count was normal. No abnormal findings were observed in routine urine tests. The hepatic and renal functions of the patient were normal. Pre-operative imaging evaluations included abdominal CT, barium swallow, and gastroscopy. The abdominal CT revealed a large low-density gastric artifact in the lower mediastinum—giant hiatal hernia (Fig. 1a). The barium swallow esophagogram confirmed the presence of a giant hiatal hernia (Fig. 1b). The gastroscopy also found the presence of it.

**Fig. 2:**
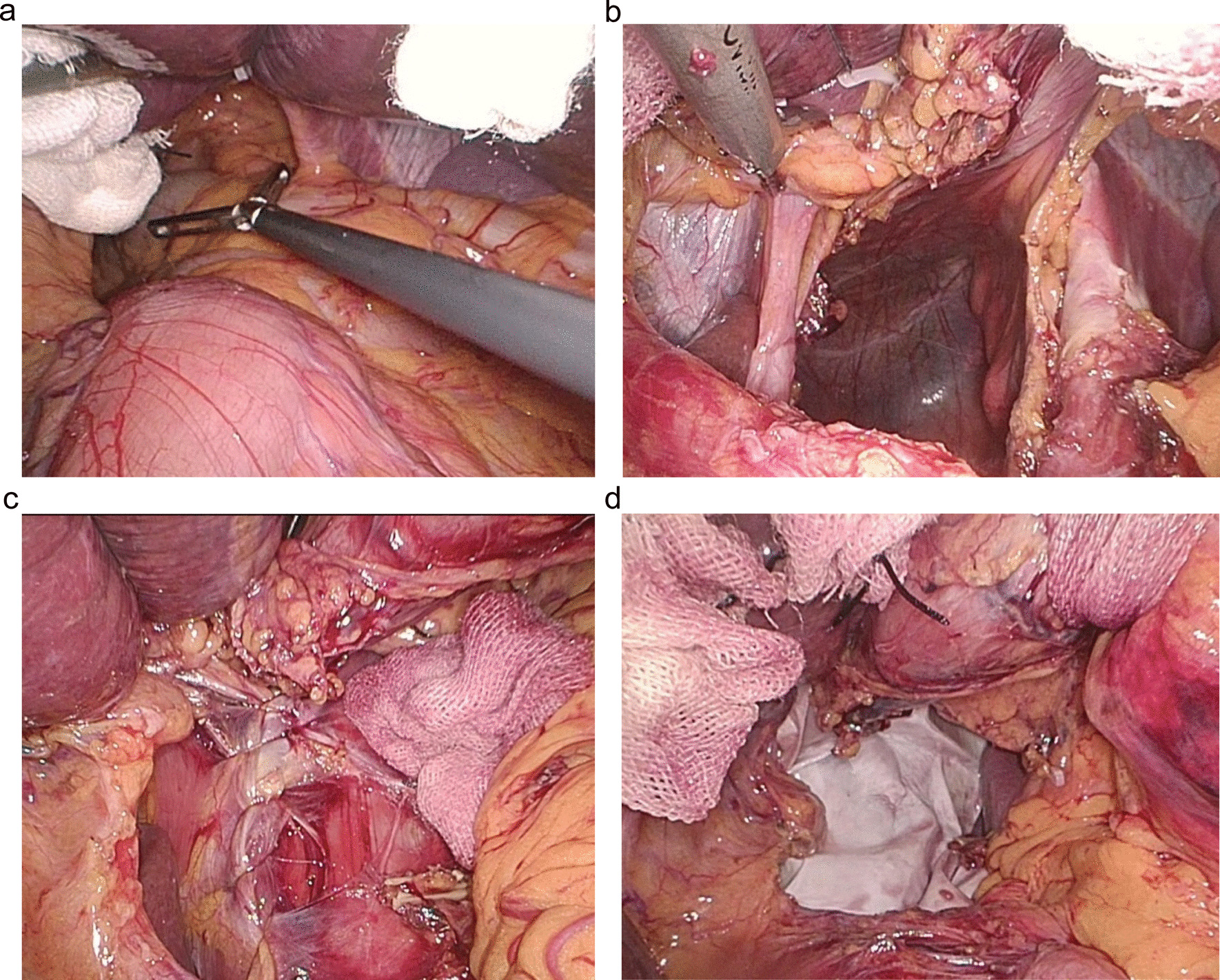
The intra-operative pictures of laparoscopic giant hiatal hernia repair. **a** The entire stomach, greater omentum and partial transverse colon protruding into the thoracic cavity. **b** All of them were sent back into the peritoneal cavity. **c** Suturing the defective diaphragm continuously. **d** Mesh reinforcement

In light of these findings, the patient underwent the transabdominal laparoscopic hiatal hernia repair with Nissen fundoplication. The procedure was carried out in the supine position under general anesthesia. After establishing pneumoperitoneum and inserting trocars into the abdomen through common four ports, standard diagnostic exploration was performed. A giant hiatal hernia was detected with the entire stomach, greater omentum, and partial transverse colon protruding into the thoracic cavity (Fig. 2a). After freeing the adhesion between the omentum and diaphragm, the entire stomach, greater omentum, and partial transverse colon were sent back into the peritoneal cavity (Fig. 2b). The diaphragm was continuously sutured with V-LOC Stitches (Fig. 2c). Then a 12 × 8-cm rectangular biologic mesh was prepared in a keyhole configuration. The biologic mesh in a keyhole was placed around the lower esophagus in the abdomen, covering the whole hiatus (Fig. 2d), which was fixed with medical glue. A 360-degree Nissen fundoplication was done to construct a wrap that was approximately 2 cm long. The procedure was well tolerated by the patient. Her right epigastric pain alleviated obviously on the first postoperative day.

**Fig. 3:**
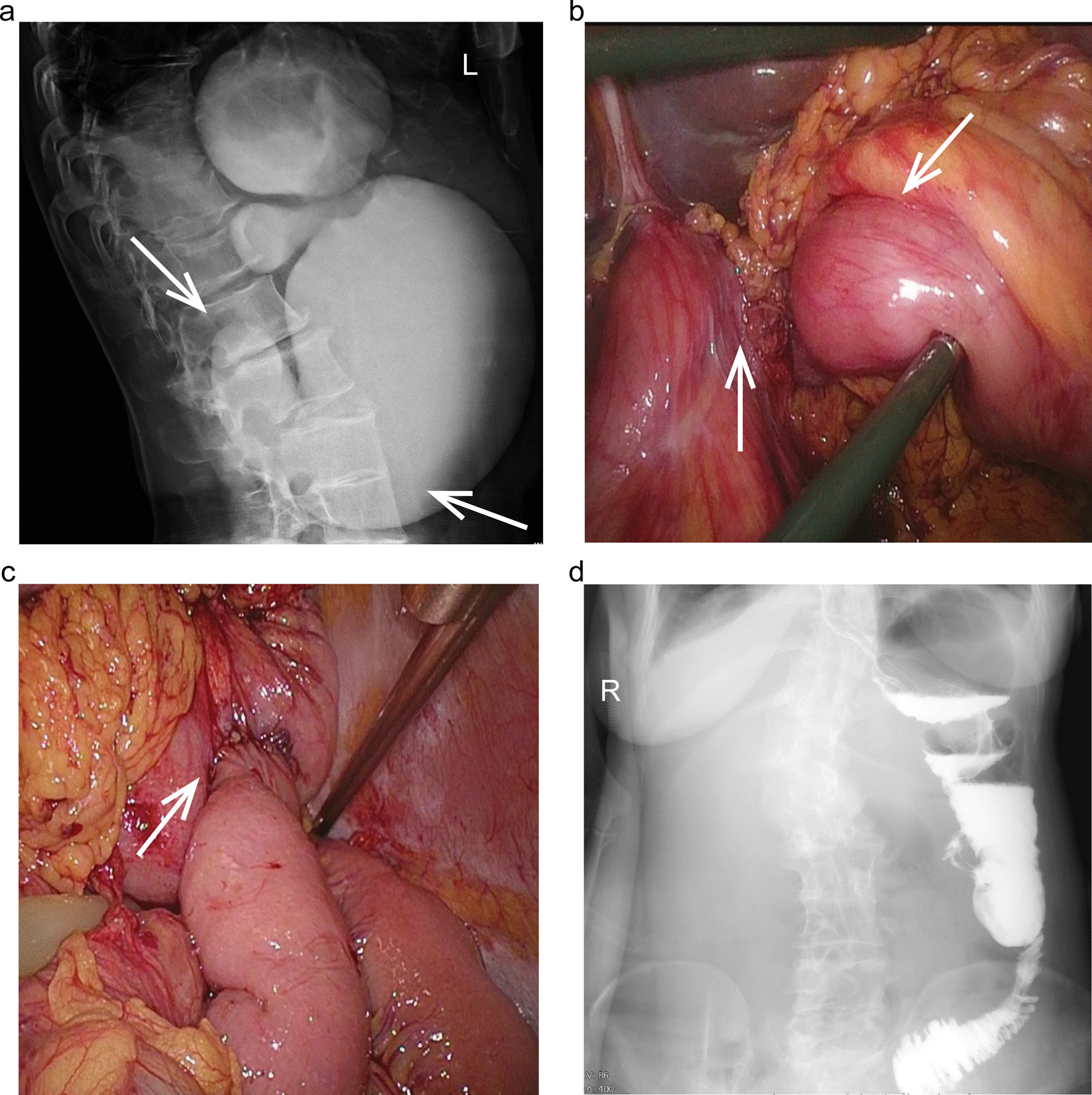
Imaging evaluations and operative pictures of post-operative gastric outflow obstruction. **a** Barium swallow showing the distension of the stomach, gastroptosis and enfoldment in duodenal bulb (white arrows). **b** Laparoscopic view of the distension of the stomach, gastroptosis and enfoldment in duodenal bulb (white arrows). **c** Laparoscopic view of the gastro-jejunal anastomosis (white arrow). **d** Barium swallow showing the gastrointestinal tract smooth (black arrow)

On the fifth day after surgery, however, the patient was presented with nausea and vomiting independent of meals. The nasogastric tube was inserted and kept in the stomach for 7 days, at the same time, the patient received nutrients intravenously. At that time, she had no nausea or vomiting. After the nasogastric tube being removed, severe nausea and vomiting of the patients occurred again. Barium swallow showed gastroptosis and enfoldment in the duodenal bulb (Fig. 3a). The symptoms mentioned above arose from gastric outflow obstruction. It could not relieve the obstruction with conservative treatments. So she underwent the second surgery to make the gastric outflow tract unobstructed. Then laparoscopic exploration was performed to find the distension of the stomach, gastroptosis, and enfoldment in the duodenal bulb (Fig. 3b). Gastrojejunostomy was carried out for her to resolve the gastric outflow tract obstruction (Fig. 3c). Liquid food intake was commenced on the fifth post-operative day when the barium swallow validated the patency of the gastrointestinal tract (Fig. 3d).

There was no nausea or vomiting for her from then on. She was successfully discharged on the tenth day after the operation. The follow-up through telephone at regular intervals was done for her. She remained asymptomatic at subsequent 1-year follow-up and was satisfied with her status.

## Discussion and conclusions

Giant hiatal hernias are relatively rare entities, accounting for 5–10% of all hiatal hernias. Although the etiology of the hiatal hernia is unclear, it can be speculated that it may be related to the following factors: increased intraabdominal pressure [4]; weakness or defect in the diaphragmatic musculature [5].

Familiar manifestations of hiatal hernias are gastric reflux, heartburn, dysphagia, nausea, vomiting, chest and epigastric discomfort [6]. As we all know, a giant hiatal hernia usually involves the stomach or other abdominal organs protruding into the left side of the thoracic cavity [7, 8] through the diaphragmatic esophageal hiatus, which always causes left epigastric discomfort. However, in this case, abdominal pain occurred in the right epigastric region, which was perhaps because abdominal organs protruded into the right thoracic cavity. The barium swallow esophagogram detected that her spine bent toward the right side, which might result in her thoracic deformity. Her thoracic deformity perhaps explained why her abdominal organs could slide into the right thoracic cavity. The study conducted by Matthew J also demonstrated scoliosis may contribute to the giant paraesophageal hiatal hernia formation [9].

Giant hiatal hernia has the potential to generate severe complications, such as visceral incarceration, strangulation, and perforation [10]. Surgical intervention is usually indicated for symptomatic patients with giant hiatal hernia [11]. The transabdominal laparoscopic procedure facilitates the reduction of the hernia sac, as well as provides better exposure and view of the intraabdominal organs. Compared to the traditional open procedure, the laparoscopic approach has similar curable effects, with the advantages of shorter hospital stay [12], faster recovery, and higher patients, satisfaction. Giant hiatal hernia repair with laparoscopic approaches should also adhere to the principles [13] of the reduction of the hernia contents, resection of the hernia sac, closure of the hiatal defect, fundoplication, and mesh reinforcement or not. In this case, the sac deeply protruded into the thoracic cavity and it adhered to mediastinal structures densely, so we did not resect the sac to avoid injury to the lung or thoracic structures.

Mesh reinforcement covering the edges of the diaphragmatic defect, decreases the recurrence of the hiatal hernia [14]. There is also literature reporting that mesh reinforcement for hiatal hernia repair [15] can cause severe complications, like esophageal erosions and stenosis. After weighing the pros and cons, we decided to use a biologic mesh to enhance the repair of the diaphragmatic defect. Fortunately, no such complications were detected in this case.

With the tacit cooperation between assistants and chief surgeon, the operation proceeded smoothly. Her right epigastric pain disappeared on the first post-operative day. However, the gastric outlet obstruction after repair of the giant hiatal hernia was unexpected. To our knowledge, cases regarding post-operative gastric outlet obstruction of giant hiatal hernia repair were rarely discussed, let alone the solutions to tackle it.

Although hiatal hernia cruroplasty and mesh reinforcement were well operated, what we neglected was that the position of the duodenum and the pancreatic head was higher than that normal position of these organs, and the capacity of the stomach was too large. With the accumulation of food in the stomach, the risk of gastroptosis and enfoldment in the duodenal bulb markedly increased, which might lead to gastric outlet obstruction. The vagal nerve injury arising from thoracic and upper abdominal operations, including hiatal hernia repair, may result in delayed gastric emptying and gastroparesis [16]. During operation, we saw omentum attached tightly to the diaphragm. So, when we freed the adhesion between the omentum and diaphragm, vagal trunks could be damaged inevitably, which might be a predisposing factor for postoperative gastric outlet obstruction. But anatomical variation mentioned above may play a principal role in this process. Nausea and vomiting were common symptoms of gastric outlet obstruction. After a period of gastrointestinal decompression, nausea and vomiting remained to occur when she had food or water again. Facing with the ineffective conservative treatments, we arranged the second operation for her. Given the reasons why the gastric outlet obstruction occurred, gastrojejunostomy was performed to allow the contents of the stomach to flow directly into the jejunum. The barium swallow after the second operation validated the patency of the gastrointestinal tract. There was no more nausea or vomiting for her since then, no matter what solid or liquid meals were taken.

In summary, facing the giant hiatal hernia repair, hiatal hernia cruroplasty, and mesh reinforcement being well operated on are insufficient, and we must watch out the anatomical variation, like the deviation of partial intra-abdominal organs from their normal positions, as well as paying attention to the protection of abdominal vagal nerve during the operation. Post-operative gastric outlet obstruction of laparoscopic hernioplasty of hiatal hernia is rare, while gastrojejunostomy can successfully relieve the gastric outlet obstruction.

## Data Availability

Not applicable.

## Abbreviation

CT: Computed tomography
